# Evaluation of Microwave Synthesis of Ceramic Pigments Based on In Situ Dielectric Characterization

**DOI:** 10.3390/ma16082976

**Published:** 2023-04-08

**Authors:** Beatriz García-Baños, Juan R. Sánchez, Jose L. Godes, Cristina Leonelli, Jose M. Catalá-Civera

**Affiliations:** 1 ITACA Institute, Universitat Politècnica de València, Camino de Vera, 46022 Valencia, Spain; 2 Al Farben S.A., Ptda. Torreta, s/n, 12110 Alcora, Spain; 3 Department of Engineering “Enzo Ferrari”, University of Modena and Reggio Emilia, Via Pietro Vivarelli 10, 41125 Modena, Italy

**Keywords:** permittivity, microwave process, pigment synthesis, in situ dielectric characterization, high temperature, ceramic pigment, mixed metal oxides

## Abstract

The application of microwave technology for efficient and environmentally friendly synthesis of ceramic pigments is a successful and rapidly evolving area of research. However, a clear understanding of the reactions and their relationship with the material absorbance has not been fully achieved. The present study introduces an in situ permittivity characterization technique, which serves as an innovative and precise tool for assessing the microwave synthesis of ceramic pigments. Several processing parameters (atmosphere, the heating rate, raw mixture composition and particle size) were evaluated by studying the permittivity curves as a function of temperature to elucidate their effect on the synthesis temperature and the final pigment quality. The validity of the proposed approach was verified through correlation with other well-known analysis techniques, such as DSC or XRD, providing valuable information about the reaction mechanisms and the optimum conditions for the synthesis process. In particular, changes in permittivity curves were linked, for the first time, to undesired metal oxide reduction at too-high heating rates and could be used to detect pigment synthesis failures and ensure product quality. The proposed dielectric analysis was also found to be a useful tool for optimizing raw material composition for the microwave process, including the use of chromium with lower specific surface area and flux removal.

## 1. Introduction

Pigments are solid and insoluble substances employed to give a medium color and/or protection [1]. Inorganic pigments based on mixed-metal oxides (MMOs) are a subcategory formed by a group of two or more metals and oxygen, with typical structures being rutile, hematite or spinel [2]. Metals commonly employed (in MMOs) are cobalt, iron, chrome, tin, antimony, titanium, manganese and aluminum. Iron oxides are the most widely used for pigments due to their non-toxicity, high stability, low price, and capacity to provide a range of colors from yellow, orange, red and brown to black [3,4]. In the context of the ceramic and plastic industries, chromium black hematite (Fe,Cr)_2_O_3_ pigment is the most extensively used, mainly in porcelain tile production, as a coloring agent in glazes, ceramic bodies and porcelain enamels [4]. This type of MMO has attracted substantial attention not only for its use as a pigment but also for its practical applicability in other important areas, such as solid catalysts, magnetic compounds, thin films or electrode materials [5,6].

Pigments based on MMOs are manufactured by calcining the mixture of appropriate metal precursor materials at temperatures ranging from 800 °C to 1300 °C, creating stable bonds that provide high chemical stability and durability to the final pigment. Temperatures are so high to improve the mobility and reactivity of the chemical species since the reactants are in a solid state [1]. The requirements of high temperature, energy consumption and long processing time lead to a trend towards faster and lower energy-demanding methods for their synthesis, being the object of intensive scientific research [4,5,7]. The objectives of these studies are mainly to reduce pigment production costs and minimize environmental damage [5].

Microwave technology application in the synthesis of ceramic pigments is gaining attention due to its fast, selective and volumetric heating, with benefits over more conventional heating techniques: enhancement of synthesis rate, homogeneous heating or lower temperatures of synthesis [8,9]. In the microwave process, the material is directly heated by electromagnetic radiation through energy conversion rather than by heat transfer by convection from thermal sources [1,10]. In addition, if the electricity that supplies the microwave system comes from renewable sources, microwave heating can reduce the typically high carbon footprint of the pigment sector to a minimum [1].

Reported studies proved that microwave synthesis accelerates the kinetics of solid-state reactions and has been successfully applied in the synthesis of many MMO pigments: yellow praseodymium-doped ZrSiO_4_, turquoise vanadium-doped ZrSiO_4_, red chromium-doped YalO_3_, black CoFe_2_O_4_, coral-red ZrSiO_4_ and many others [8,9,11]. For example, Khattab et al. reported that, in the case of Co-doped MgAl_2_O_4_ spinel, the microwave process reduced the total synthesis time to a few minutes and led to homogeneous heating of the reaction mixture in contrast to conventional furnaces [12]. Wang demonstrated similar advantages for the case of microwave hydrothermal synthesis of CoAl_2_O_4_ [13]. Other available examples of effective microwave synthesis include pigments such as Al_2_O_3_/Cr_2_O_3_ and CeO_2_:Pr pink pigment and several oxides of transition metals: Co–Fe, Ni–Fe and Fe–Fe spinels [9], showing that microwave routes are a rapidly developing area of research [14,15,16].

Although these studies reported successful preparation of intended pigments, understanding of the microwave-driven reactions is still unclear and speculative [1,14]. It is well known that the performance of pigments significantly depends not only on the microstructural parameters of precursors but also on the preparation methodology [8,17]. In the case of iron oxide pigments, their stability has been proven to depend on parameters such as particle size or shape [18]. Sometimes, undesired by-products are obtained due to homogeneity problems, nanoparticle agglomeration or uncontrollable stoichiometry due to unexpected nanometer effects [19]. Recent studies have analyzed the opportunities and challenges of microwave technology, emphasizing the need for advanced research and engineering, especially to support the design of efficient applicators and to help the control devices [1,20,21].

The main parameter that describes the microwave–matter interactions is the material permittivity. It is a complex number whose real part (dielectric constant) quantifies the capacity of the material to be polarized by an electric field. Its imaginary part (loss factor) represents its capacity to convert the energy into heat.

Electromagnetic energy and materials interact through dielectric dissipative mechanisms resulting from the polarization of material charges by the external electric field. This polarization gives rise to several types of polarization mechanisms, including (i) electronic (modification of the electrons’ position around the nucleus), (ii) atomic (positional shifts of nucleus within the molecule), (iii) orientation of dipoles and (iv) spatial charge polarization (when free electrons are confined on a surface). The dominant polarization mechanism is dependent on the frequency of the electric field [1,22].

Another key factor affecting permittivity is the temperature of the material. Recent investigations have demonstrated that the permittivity of a particular sample may differ significantly depending on the method of heating employed, whether conventional or microwave-based [21].

Additionally, the permittivity provides information about other important parameters to ensure adequate microwave processing conditions: the penetration depth and the risk of thermal runaway [1]. The penetration depth quantifies the attenuation of the electric field from the surface to the core of the sample. It determines the potential for uniformly heating the bulk material while avoiding thermal gradients near the surface. Additionally, the permittivity’s temperature-dependent behavior provides insight into the likelihood of generating hot spots that could result in undesirable thermal runaway or uncontrollable localized temperature elevation.

With these considerations, an accurate and in situ determination of permittivity as a function of pigment temperature measured under microwave conditions is crucial to assist the synthesis process design and to understand the coupling between pigment reactions and microwave field. For example, Ramos, Li, et al. addressed the design of microwave applicators for the production of ceramic pigments, relying on accurate and temperature-dependent values of pigments permittivity and emphasizing the need for these tools to gain insight on the coupling between microwave fields and heat transfer [1,23]. However, permittivity data at temperatures up to 1000 °C are very scarce in the literature since appropriate microwave measurement techniques have only recently been developed [24,25].

This work proposes using in situ permittivity determination to study the effect of different parameters on the microwave processing of ceramic pigments. The dielectric curves are correlated with other analysis techniques, such as DSC-TG or XRD. This new approach is undertaken with an aim (1) to evaluate different process parameters on the microwave reactions with a precise, fast, convenient and effective analysis technique, (2) to characterize the pigments and provide information that could be employed in numerical studies to guarantee the efficiency, repetitivity and safety of microwave pigment synthesis, and (3) to provide new insights about the relationship between synthesis parameters and microwave reactions to help solve operation and technical challenges of a potential microwave-driven industrial process.

## 2. Materials and Methods

Chromium black hematite was chosen in this study as it is a widely used reference pigment for coloring ceramics and glazes due to its high-temperature resistance, chemical stability and suitable optical properties. It has gained recent attention because of the rise in the price of black cobalt-bearing pigments and its associated environmental pollution issues [4]. It is an inorganic pigment that consists of iron (III) and chromium (III) oxides fired at high temperatures to obtain a hematite structure:

(1) $${XCr}_{2}O_{3}+{(1-X)Fe}_{2}O_{3}\overset{1000{}^{\circ}C–1050{}^{\circ}C}{\to }({Fe}_{1-X},{Cr}_{X}{)}_{2}O_{3}$$

Its composition may include boron oxide as a flux to promote the species’ mobility, reducing the reaction temperature [1].

The raw material mixtures were provided by Alfarben S.A., considering *X* = 0.3 and two types of chromium with different specific surface areas (chromium A and B with specific surface areas 5 and 2, respectively) to evaluate the effect of the particle size. This parameter is also of interest because the pigment’s particle size affects its color, brightness, transparency and the stability of the dispersion [26].

**Figure 1:**
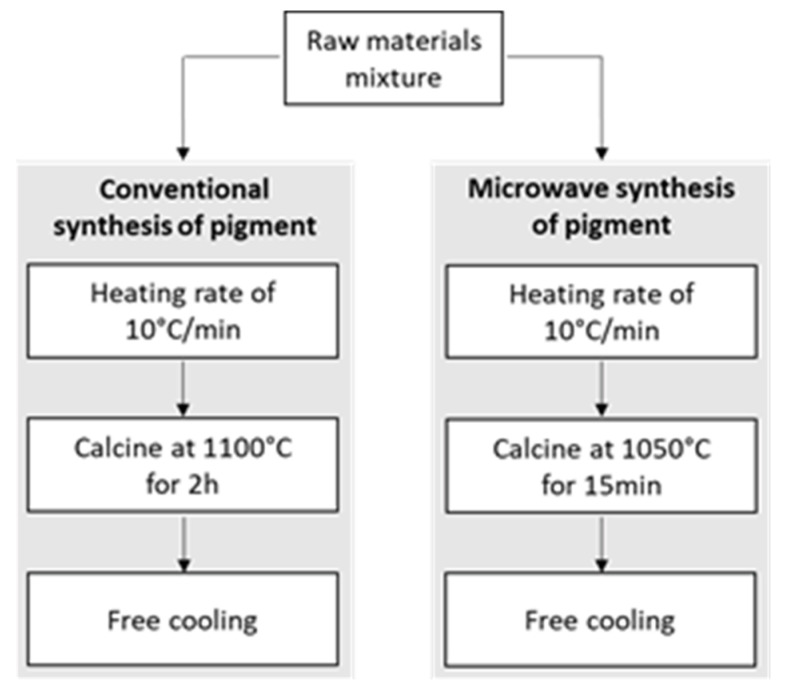
Procedures followed for the pigment synthesis: conventional and microwave-driven.

The conventional method to produce the pigment included calcinating the raw mixture in air at a high temperature (1100 °C) for approximately 2 h (see Figure 1). This pigment will be denoted as the “reference pigment”, owing to its suitability as a coloring agent validated by the manufacturer. In contrast, the protocol for synthesizing the material using microwave heating involved subjecting the raw materials to a temperature of 1050 °C for 15 min (see Figure 1). Optimizing pigment production is a critical aspect of the manufacturing process. In this regard, identifying alternative protocols, such as the microwave-based approach proposed in this study, assumes significance in expediting production and reducing costs compared to conventional protocols, as already verified by other researchers [9].

The microwave-processed samples were produced in a dual setup able to simultaneously heat and characterize the permittivity of the pigment in real-time during the synthesis [24]. The pigment was heated at a frequency close to 2.45 GHz (ISM band -Industrial, Scientific, Medical-) in a cylindrical cavity with the electromagnetic mode TE_111_. This mode provides uniform energy distribution at the center of the cavity, where the pigment sample was placed. The exact resonance frequency of the TE_111_ mode depends on the dielectric properties of the pigment, which continuously change during the heating cycle due to the increasing temperature and the different reactions. The raw mixture powder was inside a quartz tube (transparent to microwave radiation), filling a volume of 10 mm diameter and 15 mm height. The bulk temperature of the sample was calculated from the quartz holder surface measurements made by a pyrometer, after a thorough calibration process described in [27]. The microwave signal employed for heating the sample was generated by a vector network analyzer (VNA) (Rohde & Schwarz ZVRE) followed by a solid-state amplifier (RCA2026U50, Rfcore Ltd., Seongnam, Republic of Korea) in a frequency range between 2.2 GHz and 2.6 GHz, and a maximum power of 150 W. The adjustment of processing parameters as the heating rate was possible with the PID algorithm included in the control software. A detailed description of the microwave setup can be found in [24].

The raw mixture and synthesized pigments were subjected to various characterization studies described below.

### 2.1. X-ray Diffraction (XRD)

Samples of raw materials mixture and processed pigments were grinded with a porcelain mortar pestle. After that, the crystalline phases identification was carried out using X-ray Diffraction (XRD) in a fast PANalytical Cubix diffractometer (Malvern Panalytical Ltd., Cambridge, United Kingdom) by using CuKα1,2 radiation with a wavelength of λ = 1.3923, and an X’Celerator detector (Malvern Panalytical Ltd., Cambridge, United Kingdom). XRD patterns were recorded in the 2θ range from 20 to 50° and analyzed using the X’Pert HighScore Plus software (v5.1).

### 2.2. Differential Scanning Calorimetry–Thermogravimetry (DSC-TG)

Differential scanning calorimetry (DSC) and thermogravimetry (TG) were selected to study the conventional production of the pigment. In general, TG gives information about the mass loss of the sample as a function of temperature, whereas DSC is employed to detect thermal reactions. These thermal analyses were performed with a NETZSCH STA 449F5 in an air atmosphere with a flow of 50 Ml/min. Approximately 40 mg of sample was placed in a platinum crucible on an alumina pan at a heating rate of 10 °C/min, from 400 °C to 1250 °C.

### 2.3. Permittivity Characterization

The microwave-produced pigment was characterized through in situ permittivity measurements in the same set-up employed for microwave heating. The sample placed in the cylindrical cavity was measured at a frequency close to 2.45 GHz using the TM_010_ mode with a cross-coupling filter to avoid interferences with the heating mode. During the heating cycle, the sample permittivity was continuously determined from measurements of the cavity resonance parameters applying an improved perturbation method described in [24]. The reported accuracy of this method is 3% for the measurement of the dielectric constant and 10% for the loss factor.

## 3. Results

**Figure 2:**
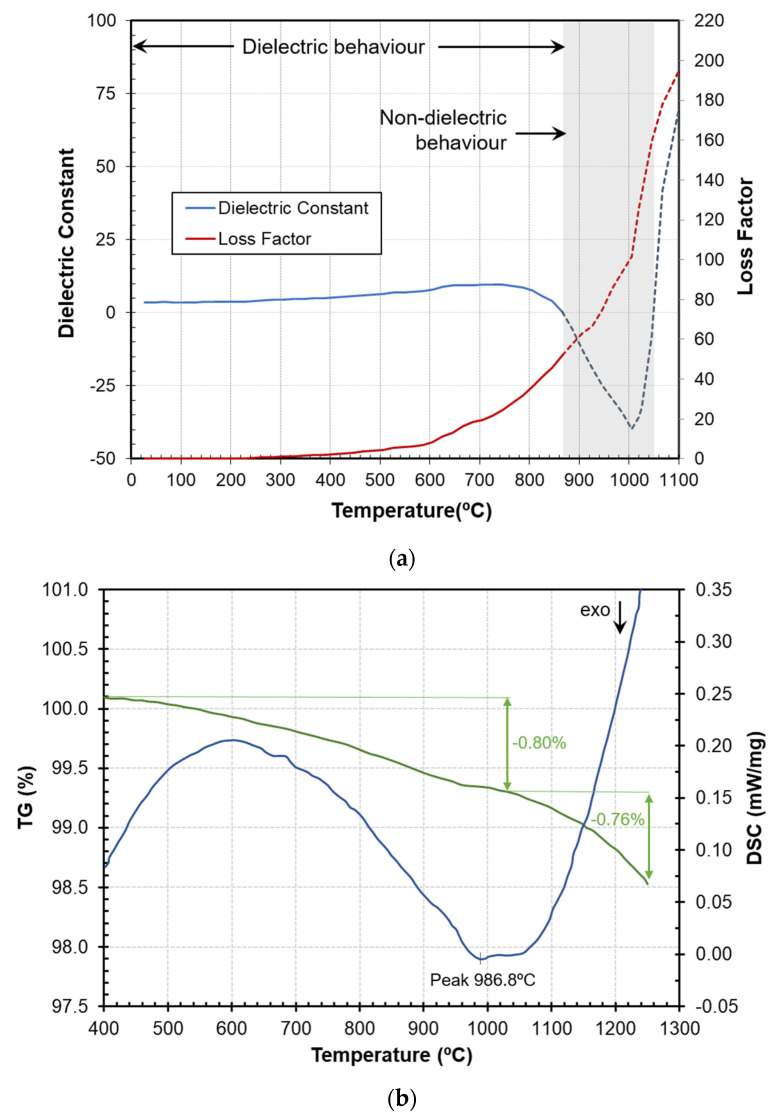
(**a**) Permittivity measurements of the raw materials mixture during the microwave synthesis of the pigment in air; (**b**) DSC-TG analysis of the same process under conventional heating.

Figure 2 shows the variation of permittivity of type A chromium black hematite (with flux) as a function of temperature during the microwave synthesis process in air atmosphere. The synthesis was performed using an average microwave power of 150 W and a heating rate of 10 °C/min, allowing an efficient and controlled pigment heating. Figure 2b displays the results of the DSC-TG experiment by conventional heating using the same heating rate as in the microwave synthesis to provide a direct comparison between the two methods. The correlation between the two techniques, even though they rely on different heating mechanisms, offers valuable information about reactions occurring within approximate temperature ranges.

Permittivity measurement at room temperature (dielectric constant 3.61, loss factor 0.038) indicated a moderate ability of the mixture to absorb microwave radiation. As the sample temperature increased, the dielectric constant and loss factor gradually increased. However, from approximately 600–650 °C the dielectric constant showed a change of trend, which correlated well with the endothermic peak observed in the DSC plot, leading to a steeper increase of the microwave absorbance up to moderate and high values (loss factor > 10) from this temperature.

Continuous heating to higher temperatures resulted in a rapid increase of the absorption of microwaves in the material, which was displayed as a rapid increase in the loss factor but a gradual decrease in the dielectric constant, becoming negative beyond 866 °C. The measured dielectric properties included contributions from both dipoles/ions and conductivity. In some materials, such as metal–insulator compounds, microwave heating can cause the metal particles to form a percolation network, connecting the entire material body and making it conductive. This leads to changes in the electrical properties of the material, and its permittivity becomes negative [28,29].

Despite being located in the region of negative permittivity, the temperature-related variations in dielectric properties exhibited a distinctive peak around 1000 °C. This peak was well correlated with the pigment synthesis, evidenced by the exothermic peak observed in the DSC curve (Figure 2b). The DSC curve had a positive slope from this temperature because the pigment structure was already formed, and less heat was needed to increase the material temperature. This was also illustrated by the slope change in the dielectric constant from this temperature. After the synthesis, the dielectric properties gradually returned to positive permittivity values and typical dielectric behavior.

Above approx. 1050 °C, hematite that had not reacted began to reduce according to the following reaction:

(2) $${Fe}_{2}O_{3}\overset{1050{}^{\circ}C}{\to }2FeO+1/2O_{2}$$

This reaction caused a change in the electrical properties of the material, leading to higher values of dielectric properties. This is in accordance with our previous studies that showed that FeO’s dielectric values are higher than those of Fe_2_O_3_ [30].

The TG showed a slight total weight loss (approx. 1.5%) that could be ascribed to some moisture or volatile compound that could be present in the raw material.

**Figure 3:**
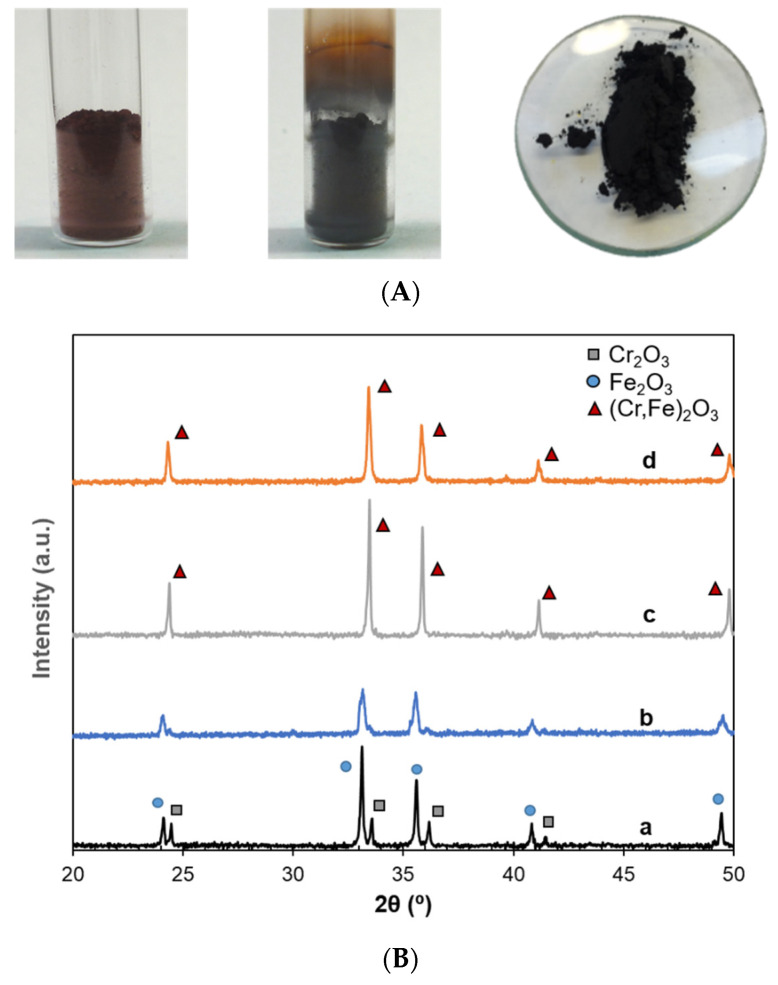
(**A**) Left: raw material mixture; Center: pigment obtained after microwave process, Right: pigment obtained after conventional process (reference pigment); (**B**) XRD patterns of a: raw material mixture, b: microwave-processed sample at 850 °C, c: microwave-processed sample at 1050 °C and d: reference pigment.

Figure 3A depicts images of the raw materials mixture before and after being subjected to a temperature of 1050 °C for 15 min within the microwave setup. The observed color change in the mixture is in accordance with the formation of the black pigment and similar to the reference pigment.

In addition, to verify the synthesis temperature and to check the pigment quality, XRD patterns of the mixture processed with microwaves at different temperatures (850 °C and 1050 °C) were compared to the raw materials and the conventional pigment. Figure 3B shows this comparison, where XRD patterns revealed the expected composition of the raw materials, with chromium and iron oxides (Figure 3B (a)). At an intermediate temperature (850 °C), peaks were wider and less defined (Figure 3B (b)), which can be ascribed to the initial formation of the pigment (hematite structure (Fe,Cr)_2_O_3_), while iron oxides are still present. The XRD curves at 1050 °C clearly show well-defined peaks of (Fe,Cr)_2_O_3_ structure, regardless of the heating method (Figure 3B (c) for microwave and Figure 3B (d) for conventional heating).

Table 1 presents the phase composition of the different samples. This table shows that, although the required processing time was substantially lower (15 min of dwelling time instead of 2 h), the microwave method did not cause any difference in the synthesis temperature or in the obtained final composition.

### 3.1. Effect of the Atmosphere

It is well known that the atmosphere’s composition plays a crucial role in the quality of the pigment. Conventional synthesis is performed in an air atmosphere, which is adequate to support the required reactions. To determine if the effect of a non-adequate atmosphere can also be identified by real-time monitoring of the permittivity, two experiments were carried out with the same conditions in air and nitrogen atmospheres.

**Figure 4:**
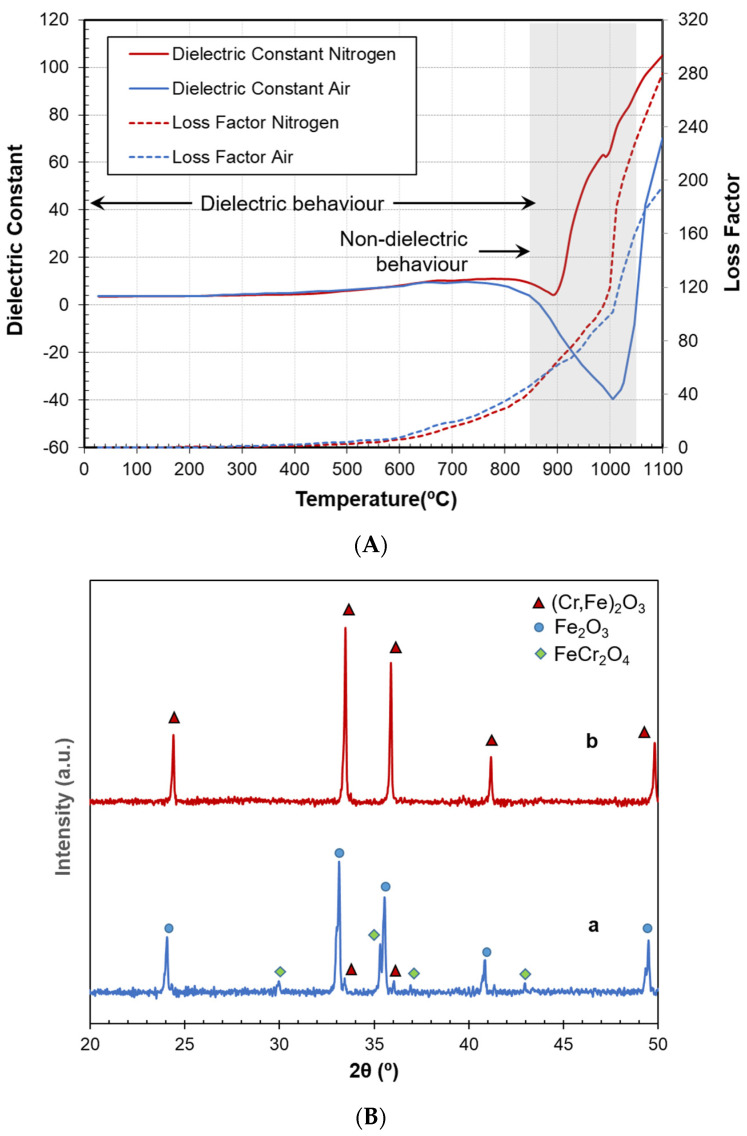
(**A**) Permittivity measurements of the raw material mixture during the microwave synthesis of the pigment in air and nitrogen atmosphere; (**B**) XRD patterns of both processed pigments. a: Microwave-processed sample in nitrogen, b: microwave-processed sample in air.

Figure 4A shows the permittivity values of both samples, with similar trends but with clear differences in the reaction temperatures and the final measured values. From the curves, the atmosphere had negligible influence below 700 °C, whereas it had a notable effect on the reactions above this temperature since the dielectric constant peak occurred at approx. 900 °C in the case of nitrogen atmosphere, well below the 1000 °C needed for correctly synthesizing the pigment obtained in air. Additionally, a difference was observed in the loss factor, mainly above 1000 °C, where the sample in nitrogen showed a greater capacity to absorb microwaves with respect to the standard pigment. This could be attributed to an undesired reduction of iron oxide in the case of the nitrogen atmosphere. This hypothesis was verified through XRD analysis of the obtained products (see Figure 4B). Indeed, the reduction of part of the iron oxide and subsequent reaction with the chromium oxide lead to chromite (FeCr_2_O_4_) formation in the nitrogen atmosphere, following the reactions:

(3) $${Fe}_{2}O_{3}\to 2FeO+0.5O_{2}$$

(4) $${FeO+Cr}_{2}O_{3}\to Fe{Cr}_{2}O_{4}$$

As expected, only a small amount of the desired product ((Fe,Cr)_2_O_3_ hematite) was observed in the sample processed in nitrogen, in contrast to the pigment processed in the air atmosphere. This could also be related to the lower magnitude of the synthesis peak shown by the dielectric constant in nitrogen. Thus, the temperature and dielectric values of the synthesis peak provided information on how the atmosphere affects the reactions. Additionally, the lower quality of one of the processed pigments could be anticipated from the magnitude of the synthesis peak in the dielectric curve.

### 3.2. Effect of the Heating Rate

**Figure 5:**
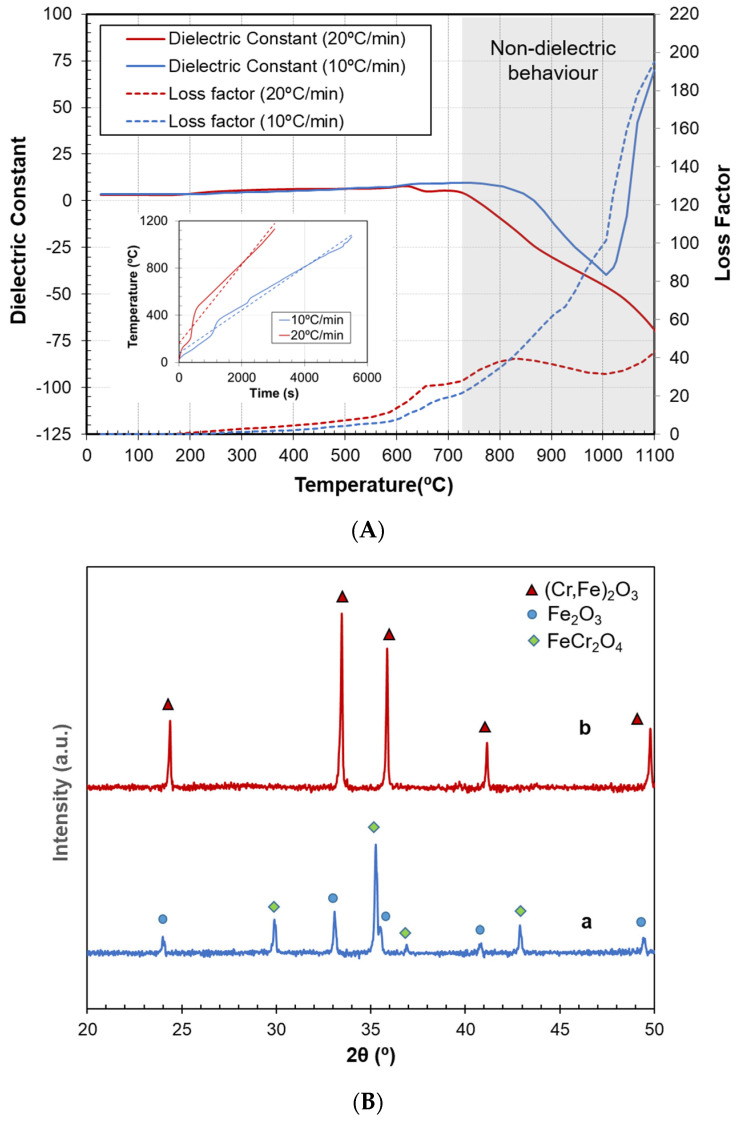
(**A**) Permittivity measurements of the raw material mixture during the microwave synthesis of the pigment at two different heating rates (10 °C/min and 20 °C/min). Inset: heating rates applied during the process; (**B**) XRD patterns of both processed pigments. a: Microwave-processed sample at 20 °C/min, b: microwave-processed sample at 10 °C/min.

Another parameter that severely affects the synthesis process is the heating rate. A heating rate of 10 °C/min is usually applied in the conventional process to obtain the adequate crystalline structure. However, microwave heating is widely known to produce faster reactions, and researchers often evaluate the possibility of increasing the heating rate, sometimes leading to undesired reactions. To study this effect on the dielectric curves, two specimens were processed at 10 °C/min and 20 °C/min, and Figure 5A shows the permittivity of both samples during the heating cycle.

According to the results, large differences could be expected in the processed products, since dielectric property curves indicated different reactions taking place. In the case of a higher heating rate, the curve started to decrease at lower temperatures, revealing that some reactions began earlier. It did not present, however, the synthesis peak observed in all the other experiments. The loss factor also attained much lower values than in previous cases. These characteristics in the permittivity curves were attributed to the absence of adequate crystalline structure formation. This was verified by the XRD pattern (Figure 5B), which did not show evidence of the (Fe,Cr)_2_O_3_ hematite. It showed, however, a higher quantity of chromite and a part of the hematite still unreacted. Thus, early changes in the permittivity curves revealed an undesired reduction of the iron oxide, which subsequently reacted with the chromium oxide to form chromite instead of the hematite structure of the pigment.

This example shows the potential of dielectric monitoring as a tool for real-time identification of undesired reactions, rather than relying on other analyses that are often post-process, destructive and time-consuming.

### 3.3. Effect of the Type of Chromium and Use of Flux

Finally, the effect of variations in the raw mixture composition was assessed by comparing the permittivity curves under microwave heating. Samples with and without flux (boron oxide) and also with different types of chromium (A and B, with specific surface areas of 5 and 2, respectively) were prepared, and the permittivity was recorded during the pigment synthesis (10 °C/min, air atmosphere). In all four cases, it was verified that the correct pigment was formed by identification of the proper phase composition in XRD (results not shown, identical to those presented in Figure 3B (c) and Table 1).

**Figure 6:**
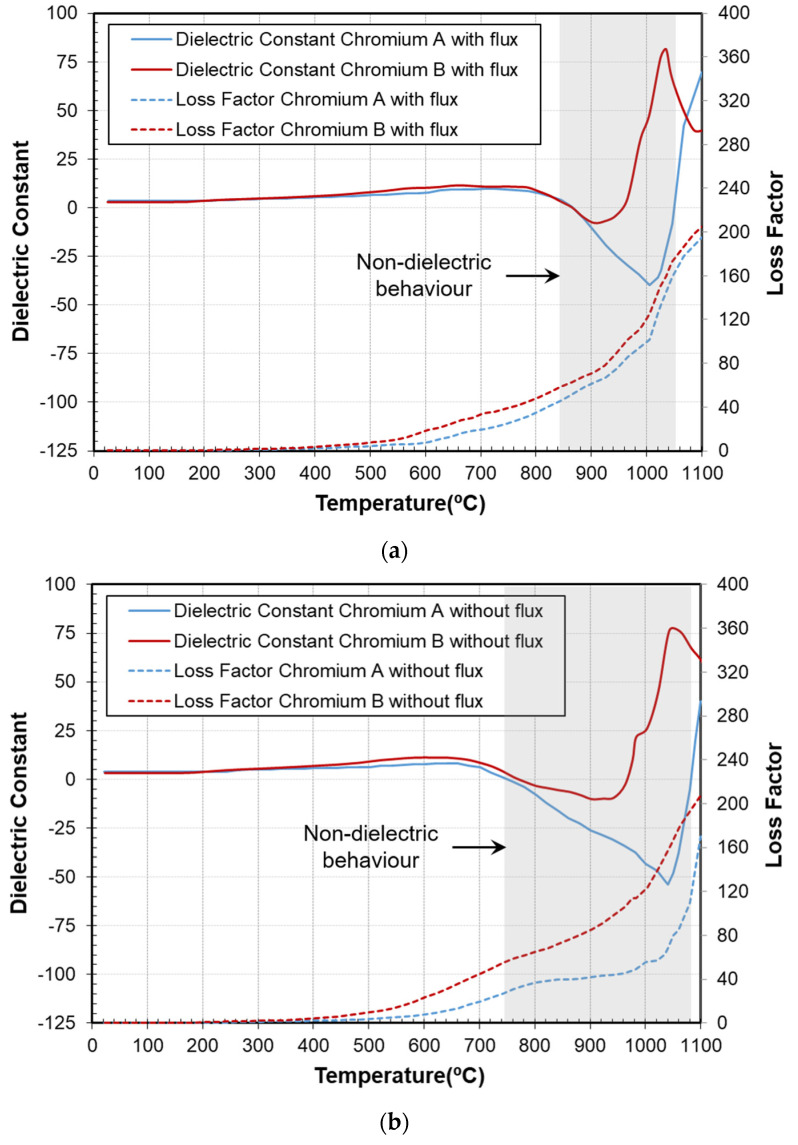
Permittivity measurements of the samples during the microwave synthesis of the pigment: (**a**) raw material mixture including flux (boron oxide) and with different types of chromium; (**b**) raw material mixture without flux and with different types of chromium.

Figure 6 compares the four samples, in Figure 6a including the flux and in Figure 6b without the flux. The behavior of the dielectric curves was similar for all of them, reaching similar permittivity values at the end of the heating cycles. However, they showed some differences in the synthesis temperature, as reflected by the positions of the characteristic peak in the dielectric constant.

From both figures, it was inferred that pigments with chromium A required higher synthesis temperatures than those with chromium B (approx. 100 °C higher). This was expected, since particles with a higher specific surface area present more surface to be activated, requiring higher synthesis temperatures [26].

Regarding the flux, its influence on the permittivity curves was not clearly noticeable. Only in the case of chromium-A-containing samples was a decrease in the synthesis temperature from 1050 °C to 1000 °C observed. The dielectric curves demonstrate that the application of an alternating electric field prompts the species’ mobility and diminishes the necessity for flux in comparison to conventional methods.

## 4. Conclusions

In this work, an advanced in situ permittivity characterization technique was employed to study microwave-driven pigment synthesis.

This study presented, for the first time, the identification of pigment synthesis temperature from dielectric curves in real time and with high accuracy, correlated with established analytical methods such as DSC or XRD. Further studies will include the integration of microwave heating, dielectric analysis and in situ TG-DSC in a combined setup.

Changes observed in the dielectric curves provided information about the reactions during the heating cycle. For example, dielectric monitoring revealed undesired reduction of the metal oxides under a reducing atmosphere or caused by an excessive heating rate. These results illustrated the potential of this monitoring technique as a fast prediction tool to detect failures in microwave pigment synthesis and to ensure the required product quality.

The evolution of the pigment loss factor throughout the process also serves as a valuable aid for defining control functionalities in a potential industrial application. This factor identifies critical temperatures and stages in which the regulation of microwave power application is necessary to prevent runaway behavior that could result in the undesired formation of magnetite or metal iron.

Finally, permittivity determination was proven to be a reliable and convenient tool to optimize the raw material composition for the microwave process. Dielectric curves showed the convenience of using chromium B (with a lower specific surface area), and revealed the possibility of removing the flux without affecting the synthesis temperature.

Obtaining this information is crucial for optimizing the studied process, whether it is achieved by lowering the necessary synthesis temperature or by minimizing the number of components required in the mixture.

## Figures and Tables

**Table 1:** Phase composition of the different samples.

Sample	% Fe_2_O_3_	% Cr_2_O_3_	%(Cr,Fe)_2_O_3_
Raw material	70.00	30.00	0
Microwave—pigment at 850 °C	20.79	3.56	75.65
Microwave—pigment at 1050 °C	1.89	0.12	97.99
Conventional—Reference pigment	3.62	0.19	96.19

## Data Availability

Not applicable.
